# A Mobile Phone App for Bedside Nursing Care: Design and Development Using an Adapted Software Development Life Cycle Model

**DOI:** 10.2196/12551

**Published:** 2019-04-11

**Authors:** Frederic Ehrler, Christian Lovis, Katherine Blondon

**Affiliations:** 1 Division of Medical Information Sciences University Hospitals of Geneva Geneva Switzerland; 2 Faculty of Medicine University of Geneva Geneva Switzerland; 3 Medical Directorate University Hospitals of Geneva Geneva Switzerland

**Keywords:** mHealth, nursing, hospital information systems

## Abstract

**Background:**

Nurses are increasingly spending time on computers, and providing them with a tailored tool to access clinical information and perform documentation at the bedside could help to improve their efficiency. Designing an app to support nurses’ work at the bedside is a challenging task, given the complexity of the care process.

**Objective:**

This study aimed to present the design, development, and testing of a smartphone app for nurses guided by an adapted software development life cycle model that takes into consideration the complexity and constraints of a health care setting.

**Methods:**

The model drives us through an iterative development process intersected by 3 stages of formative evaluation of growing ecological validity.

**Results:**

The initial requirements identification stage included 11 participants who helped us select the most important functionalities to integrate into the tool. Starting with a usability evaluation allowed for the identification of design issues that could have caused misuse. Then, making on-site evaluations under the supervision of an investigator helped to understand the adequacy of the tool with limited risks. Finally, the on-site evaluation allowed us to validate the acceptance of the app by caregivers.

**Conclusions:**

The interpretation of the collected evaluation confirms the necessary involvement of end users early in the process to help address the heterogeneity of the nursing workflow processes in the different wards. We also highlight the delicate balance between high-security measures to protect access to patient data and maintaining ease of access for efficiency and usability. Although a close collaboration with clinicians throughout the entire project facilitated the development of a tailored solution, it was also important to involve all stakeholders, in particular, the information technology (IT) security officers.

## Introduction

### Background

Studies on workplace efficiency and patient care have brought forward the need to improve workflow processes [1], particularly with the implementation of electronic health records (EHRs) and documentation requirements. Nurses and nursing assistants provide a range of interventions for each patient, which are determined through planned nursing care or doctor prescriptions. For example, they administer medications, check vital signs, change bandages, handle meals, and provide patient support and education. All these bedside activities vary for each patient. In our hospital, nurses print out the intervention list for each patient at the beginning of each shift to guide the provision of care. This list is also used as support for patient handoffs at the beginning of the shift and helps nurses prepare the necessary equipment for patient care before entering the rooms. Throughout the shift, nurses document assessments such as vital signs, observations, as well as the planned interventions.

Although the printed task lists guide the bedside activities, they are also potential sources of errors. Printouts are snapshots of patient interventions at a point of time, and updates in the EHR need to be scribbled in manually and may even be missed. Furthermore, these printouts do not replace the need for nurses to log into the patients’ EHRs to document their actions and observations [2]. These paper-based supports are also used as temporary repositories for clinical data (ie, vital signs) before they are entered into the EHR; besides delaying the availability of these data in the EHR, this transcription process increases the risk of errors [3]. Finally, the institution’s move toward going paperless is another reason to look for alternative solutions to improve the bedside patient care process.

Mobile devices provide many new opportunities and tools for patient care [4-10]. In fact, a smartphone app can help address some of the concerns raised above [11]. An EHR-connected app can provide the needed intervention list at timely moments and allows real-time documentation [12,13]. For example, entering data directly on the device at the bedside could help avoid potential transcription errors, with data immediately available in the EHR. In addition, access to elements in the EHR can help nurses respond to patient questions more readily, thus encouraging patient empowerment. Most importantly, however, we hypothesize that a smartphone app can help decrease the amount of time spent on clinical documentation and updates in the medical charts by supporting the nurses’ workflow process. Previous studies report variable time spent on documentation (20%-35% of a shift), which is often completed away from the patient [14-17].

Although several projects have already explored the use of mobile health (mHealth) for physicians [18,19], providing mobile access to EHRs is still in its budding stage. However, it is recognized that designing and developing a mobile app in the complexity of a health care environment includes many challenges linked to specific constraints of the medical domain as well as linked to the form factor of mobile apps [20,21].

Despite an accumulation of best practices research that identifies success factors, IT implementation projects are often not successful. Across industry sectors, at least 40% of such generic IT projects are either abandoned or fail to meet business requirements, whereas fewer than 40% of large systems purchased from vendors meet their goals [22]. Some sources even report failure rates of up to 70% [23].

There are serious issues with the implementation of the health information system (HIS), and reports of HIS implementation failure are not hard to find in the literature. The reasons for failure include inadequate funding; lack of IT infrastructure; poor leadership; inadequate end-user engagement and unrealistic timelines; and lack of compatibility of HIS with current work processes, the organizational culture, and vision [24-27].

### Previous Works

We experienced these difficulties during a 2014 pilot study conducted in 3 wards of our institution to explore the use of tablets with EHR access for nursing teams. The EHR version was the same as the desktop one and was used during a week-long test with workstations on wheels (WOWs). These tablets not only provided support for documentation at the bedside but also helped avoid iterative trips to the nursing station to collect the various supplies for bedside care. WOWs with laptops were also available in the wards during this period. Nurses printed less task lists, reported higher efficiency in validating tasks, and spent more time at the bedside. However, the tablet EHR did not adequately support the nurses’ workflow, for instance, it did not provide an overview of the assigned tasks, so the nurses could not plan the tasks for the day. In fact, some tasks were even forgotten or performed with a delay. Usability of EHR on the tablet was low, with difficulty reading the data due to the screen size, fonts, and the lack of adaptation of the EHR to the mobile device. Suggested improvements were the grouping of similar tasks together and providing multipatient views of the assigned tasks to organize their work. Furthermore, rethinking the design of the EHR for tablets could help address the usability issues and could help provide an improved support tool throughout the nursing workflow. This was confirmed in a study in another setting [28].

Developing mobile apps in a health care setting is not a simple task. Several approaches have been proposed that can be followed to drive the development of apps, but there is often a lack of feedback on the impact of the chosen method on the quality and acceptance of the produced app [29].

**Figure 1:**
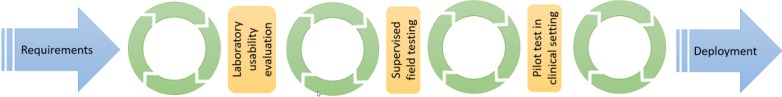
Overall life cycle process with the different evaluation stages.

As an attempt to deal with the strong constraint associated with the development of health information technology (HIT) in health care, we proposed, in a previous paper, a tailored software development life cycle (SDLC) model that takes into consideration the constraints of mobile app in a health care environment and integrates both development and evaluation frameworks (Figure 1) [30].

There is no clear consensus on existing SDLC models, but we can confidently divide them into traditional and agile models. Traditional models are mainly sequential and include models such as the waterfall, the spiral, or the V-shape. Agile models, on the other hand, are a sequence of short cycles that allow better responsiveness [31-33]. Our adapted SDLC model differs from the existing ones as it adds more detail in the development stage; several substages are separated by evaluations of increasing ecological validity.

This model begins with a requirements identification stage, followed by a series of development cycles, including specification, prototyping, development, and functional validation, interspersed by formative evaluations of growing ecological validity. Starting with low constraints in the early testing stage allowed us to validate different aspects of the tool without being limited by unnecessary complexity.

### Objectives

In this paper, we aim to demonstrate how our tailored SDLC model based on evidence and user feedback can drive the design and development process of an app in a health care setting, from the initial brainstorming to the testing of a tool connected to our local EHR system. We also describe the conceptual framework adopted to address the hospital security requirements for implementation.

## Methods

### Study Design

We recruited nurses, nursing aids, and head nurses from the departments of medicine and surgery as cocreators throughout our project. These departments have the highest number of beds for acute care in our hospital. Choosing one unit from each department aimed at being the most representative of ward work in acute care settings in our hospital. Therefore, by creating a tool that addresses their combined needs, we hoped to better anticipate a future all-hospital deployment. The objective of the app was to provide access to certain parts of the EHR, targeting the elements in the bedside workflow, which can benefit from timelier data entry into the EHR. We proceeded according to the adapted SDLC model (Table 1), starting with requirements identification, followed by subsequent iterative development and evaluation stages.

### Requirements Identification

The overall process started with the identification of the needs through a couple of focus groups with the end users. The first focus group focused on brainstorming and determining which target population (nurses, physicians, and patients) and which needs could be addressed with mobile access to the EHR. The results were subsequently analyzed in depth, using a thematic analysis approach and included the use of card-sorting, to define institutional priorities and feasibility of the various propositions, taking into consideration the available resources (ie, available data, estimated complexity, and potential gain). Card-sorting is a reliable approach to find patterns in the way participants organize content and was used to identify common elements in design from the various brainstorming ideas [34]. The subsequent analysis addressed the feasibility of each idea and led to the choice of an app for nurses at the bedside. The aim of the following focus group session was to understand the workflow of the nurses and to identify the main interactions that they have with the clinical information system. Discussions with end users were transcribed with a subsequent thematic analysis to identify the main interactions between the users and the system.

### Development Cycles

The initial requirement fueled the start of iterative user-centered development cycles. The cycle started with the creation of mockups integrating the specifications identified from the previous cycle. The mockups were discussed with the project team for validation. The validated evolutions were then implemented into the functional prototype and tested using functional evaluations. In these evaluations, all app functions were executed in a test case procedure. Then, the actual and expected outputs were compared to check whether the app addressed the specified end user needs. At each stage, the prototype of increasing complexity was presented to a group of end users to gather feedback about the proposed concepts. This feedback was integrated into the next cycle to refine the functionalities as well as the interaction through the user interface.

### Formative Evaluations

We conducted formative evaluations at key milestones of the project. These formative evaluations were more in-depth than those performed during the iterative development cycles and often raised issues that had not been noticed during the development cycles. They, thus, contributed to the evolution of the tool specifications.

**Table 1:** Summary of the software development life cycle (SDLC) stages and goal description.

Number	Stage	Methodology	Stage purpose
1	Brainstorming	Focus group	Identification of the most promising areas to develop and intervene
2	Requirements	Focus group	Identification of the basic functional requirements
3	Development cycle 1	Development cycles	Development of the Alpha prototype
4	Usability test	Laboratory usability test	Evaluation of the tool usability
5	Development cycle 2	Development cycles	Development of the Beta prototype
6	User test	Supervised on-site test	Test of the tool on site to validate workflow adequacy
7	Development cycle 3	Development cycles	Development of the final prototype
8	Pilot test	Unsupervised on-site test	Summative evaluation to test app acceptance

#### Usability Test

The first formative evaluation was a laboratory usability test [35-38]. During this evaluation, nurses performed one of the two predefined scenarios in a controlled but realistic environment. The scenarios were created according to the results of previous field observations. Overall, one scenario was designed for the medical ward nurses and one for the surgical ward nurses. The scenarios guide the participants through a sequence of actions that the nurses are likely to perform in real life and that are supported by our app.

The outline of the scenario is as follows:

1. Identify the patient by scanning the Quick Response (QR) code on his bracelet
2. Review the interventions performed during the night
3. State the necessary interventions for the medication rounds, validate the administration of drugs, and cancel the validated breakfast task
4. Postpone and duplicate an intervention
5. Validate the start of an intravenous (IV) drug; check the pro re nata (PRN) painkillers, administer a dose of painkiller, and validate this action in the app
6. Indicate the elapsed volume for the IV drug and document the patient’s pain level
7. Document that the patient refused to eat his dinner
8. Complete a Braden scale assessment and take a photo of the lesion
9. List the remaining interventions to be completed before the end of the shift
10. Log out

After a brief presentation of the tool, participants were asked to follow the scenario and to perform the sequence of tasks. All the tasks were video-recorded for subsequent analysis. We measured the success rate for the tasks and reported the errors with the proposed improvements. The participants completed the System Usability Scale (SUS) questionnaire [39] and discussed their satisfaction and impressions at the end of the session. The investigators then conducted an analysis of the usability of the tool.

#### Supervised Field Tests

Supervised field tests took place in 2 wards of the University Hospitals of Geneva (HUG). After a short personalized training, the care providers were asked to use the app to perform their daily tasks and were followed by an observer. The observer closely monitored their use of the app and identified usability issues and bugs. After each session, bugs were systematically reported to the development team for corrections. This close supervision was vital to ensure patient safety and included the verification that all patient information handled with the app was correctly recorded in the EHR at the end of each test session.

#### Pilot Test in the Wards

The pilot study took place in a surgical ward and a medical ward of 18 beds, each at the HUG [40]. The app was provided to the participants on institutional smartphones. The app usage was restricted to weekdays from 7 am to 6 pm to ensure technical support in case of problems.

During the week before the start of the study, ward nurses and nursing assistants received a short training about the app. As participation in the study was on a voluntary basis, the participants were informed that the use of the app was encouraged but not compulsory during the study period. During the whole study, the investigators conducted frequent visits to the wards to provide support and to collect feedback from the users, in particular, for bugs and suggestions for improvement. These findings were forwarded to the IT team, who corrected the bugs and added minor improvements when possible.

To evaluate the usage of the tool by the participants, the app logged all of the participants’ interactions with the app automatically, for subsequent analysis. At the end of the study, participants were asked to complete a tailored technology acceptance questionnaire. The questionnaire was derived from the unified theory of acceptance and use of technology (UTAUT) [41] to fit our particular setting. It contained 21 questions with a 7-point Likert scale, which were divided into 5 dimensions: performance efficiency (4 questions), effort expectancy (4 questions), social influence (4 questions), facilitating conditions (4 questions), and behavioral intention to use the system in the future (3 questions). Perceived usage during the study and general comments were also collected.

## Results

### Setting

Our research took place at a tertiary teaching hospital of 1800 beds, with 60,000 admissions per year and more than 4000 nurses. Table 2 presents a summary of the methods and number of participants at each stage of the SDLC model.

### Requirements Identification

#### Ideation

The initial brainstorming session with 24 nurses and nursing assistants and 8 physicians from medical and surgical wards produced 30 different ideas about how mobile devices could help address needs of nurses, physicians, and patients in our institution. Among these propositions, many required data that were not available electronically or that were not available within the EHR (eg, menu choices for patients) or had low feasibility or low priority (scan of ward pharmacy to automatically generate stock refills). After considering estimated cost and complexity as well as potential gain for the institution, the project team decided to focus on a bedside support tool for nursing teams. This was the beginning of Project *Bedside Mobility*.

#### Requirements Collection

In the subsequent focus group with 5 nurses, 4 nursing assistants, and 2 head nurse investigators, we explored in more detail the functionalities that nurses envisioned to support their workflow. The thematic analysis of the focus group transcript revealed the need to improve current processes: scattered information retrieval, photo and vital sign documentation needs at the bedside, and lack of a continuously updated task list (Textbox 1). We found that nurses spent considerable time retrieving information and entering data in the EHR because it is scattered in several sections of the EHR. Nurses wanted to be able to easily document wound progress with pictures at the bedside. Picture uploads in the EHR are a multistep process, which involved using and connecting an additional device (ie, camera) and manually entering data (ie, consent, date [current and date of photo], type of photo, etc).

We also collected suggestions about new possibilities that do not exist in the EHR (Textbox 2). First, by identifying one’s assigned patients for a given shift, one should be able to visualize the required tasks in both an individual or multipatient view. In addition, caregivers requested to simplify patient identification at the bedside. Second, users wished to simplify the process between task validation and data entry, such as for blood glucose levels or fluids (ins, and outs), rather than visualizing and validating the task list in one page and then going to another page to enter the data. They also wished to be able to receive notifications (eg, at the end of an IV drip). Finally, the nurses also needed assistance in calculations such as for fluid balance; currently, data for the fluid intake and output are collected on pieces of paper at the bedside or in the toilet facilities, and the total is calculated with a calculator before data entry in the EHR.

#### Selected Functionalities

**Figure 2:**
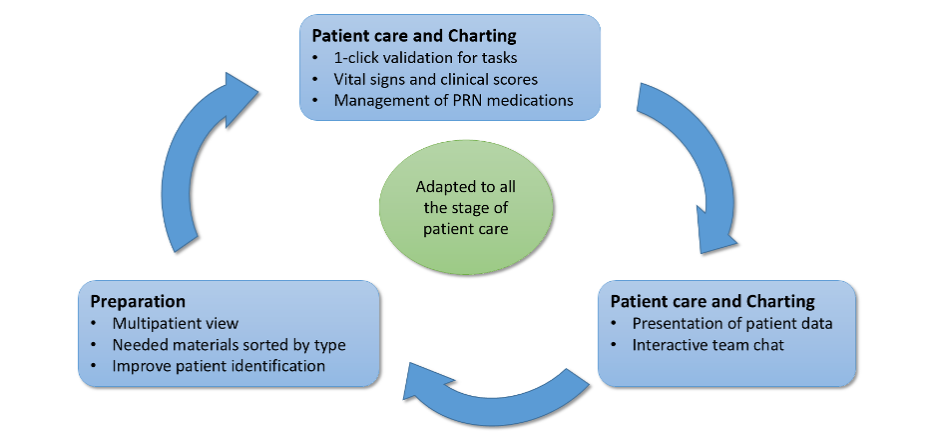
Overview of app functionalities (PRN: pro re nata).

Functionalities were selected from the initial needs assessment and were refined with iterative discussions with the nursing staff. We provide a brief overview of the app’s main functionalities in Figure 2.

In accordance with our hospital’s patient safety strategy, the app provides access to patient charts by scanning a QR code on the patients’ hospital bracelets with the smartphone camera.

Each patient chart includes general patient information data (identity, age, and length of hospital stay) with clinical data on the current hospitalization, comorbidities, and daily nursing objectives. These components were included to provide support for the handoff process.

**Figure 3:**
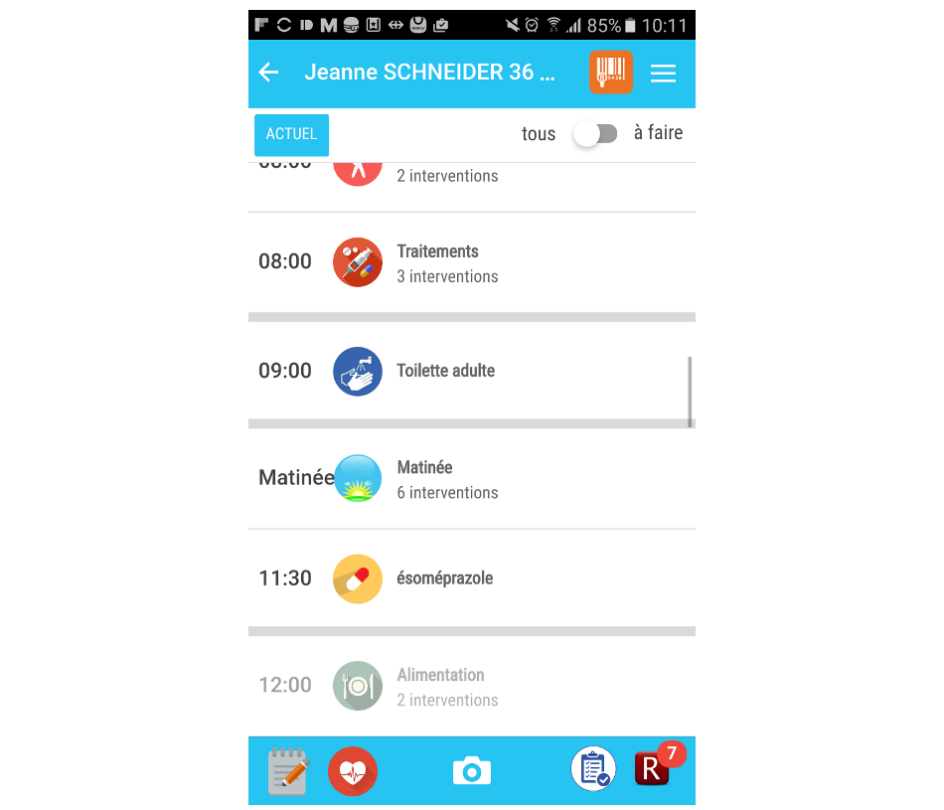
Screenshot of the Bedside Mobility app, view of the daily intervention.

In each patient’s chart, nursing interventions are sorted in a chronological order, starting from the current time of app use. Interventions of similar nature (eg, medication) are grouped together for easier readability (Figure 3). Interventions can be validated when completed with a rapid swipe motion, but can also be modified, delayed, repeated, or validated as incomplete (eg, in the case of patient refusal). These functions are often used by nurses during charting in the EHR.

**Table 2:** Summary of the software development life cycle (SDLC) stages and user involvement.

Number	Stage	Methodology	Number of participants	Iterations	Duration
1	Brainstorming	Focus group	32	1	1 month
2	Requirements	Focus group	11	2	2 months
3	Development 1	Development cycles	5	8	12 months
4	Usability test	Laboratory usability test	10	1	1 month
5	Development 2	Development cycles	5	2	4 months
6	User test	Supervised on-site test	20	1	2 weeks per ward
7	Development 3	Development cycles	5	2	1 month
8	Pilot test	Unsupervised on-site test	30	2	2 months

**Weaknesses identified in the electronic health records.**

- Scattered information
- Complicated data entry: vital signs and photos
- Lack of real-time task updates

**New desired functionalities.**

- Identifying assigned patients during a shift
- Simplified patient identification at the bedside
- Direct data entry: ins and outs and blood glucose levels
- Assistance for calculations (eg, total volumes of fluid)
- Notifications (eg, end of intravenous drip)

**Figure 4:**
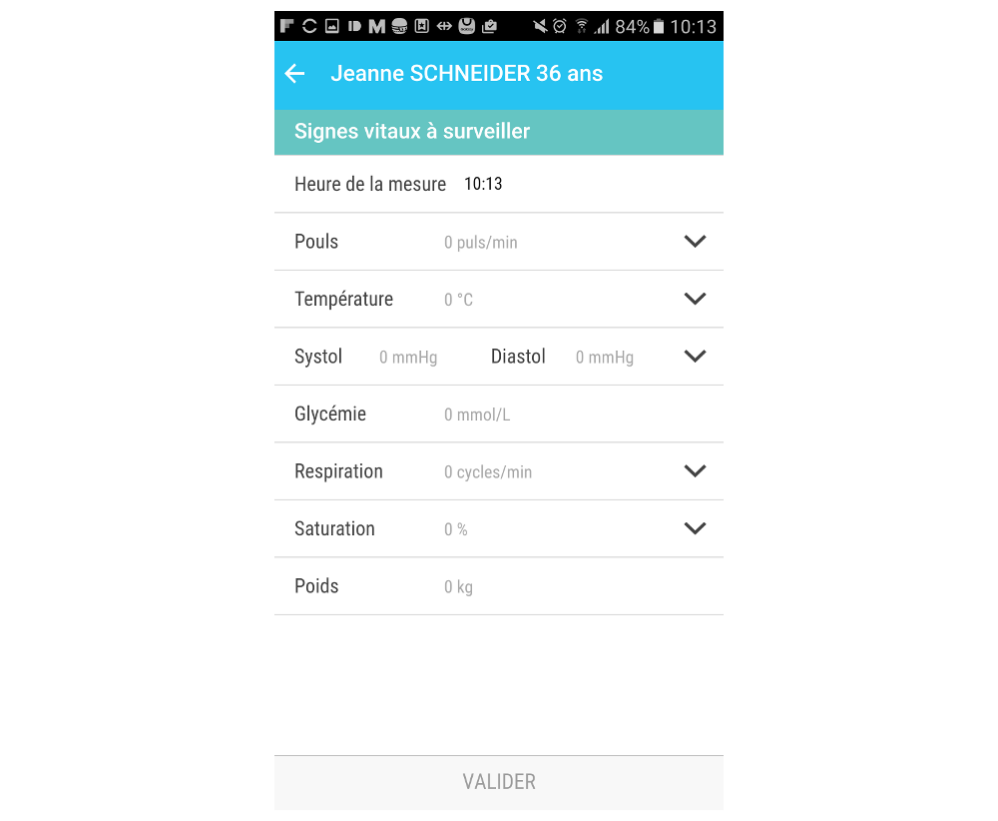
Screenshots of the Bedside Mobility app, input screen of clinical score.

**Figure 5:**
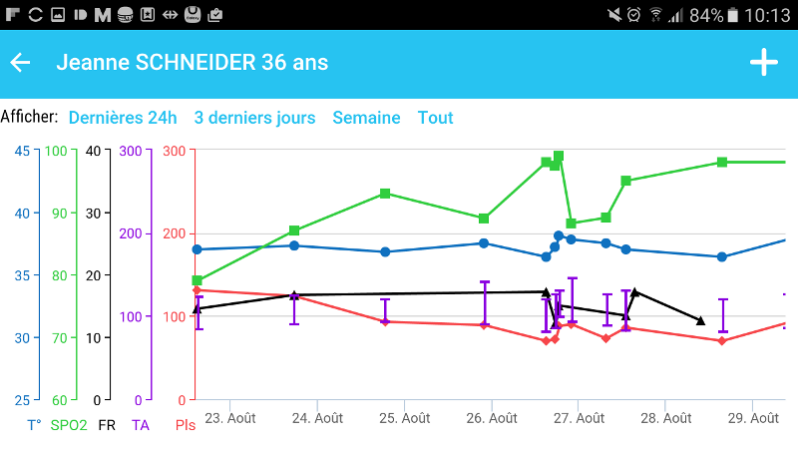
Screenshots of the Bedside Mobility app, vital signs graph.

Vital signs and clinical scores can be entered and visualized easily in the app; data can be entered directly from the task list to facilitate usability (Figure 4) when these assessments are planned. The app also allows users to enter these assessments in the data visualization screen when they are not scheduled (Figure 5). We chose to use vital sign graphs that are similar to the EHR graphs for easier readability.

PRN use medication (or medication “as needed”) is available in another part of the app, with indications of prescribed doses and frequencies. It also records when the doses are administered during the past 24 hours, as requested by the nursing teams.

A “notes” function was implemented to allow easy note taking during the day for subsequent production of progress notes, charting, or handoffs. A note can also be created from the to-do list section.

Careful consideration was given to the design of the app to provide efficiency and usability, while offering the range of frequent tasks used in clinical documentation from a computer-based EHR.

### Development Cycles

#### Technical Considerations

From an architectural point of view, we set up a client-server architecture. Our server, hosted on a Java Beans Open Source Server of the hospital infrastructure, is programmed in JAVA and is responsible to ensure communication between the app and the exposed services of our local EHR. The communication is proprietary using Representational State Transfer (REST) or XML messages and allows access to all the necessary information in a standardized way. Between our server and the client, a proprietary REST or JavaScript Object Notation exchange protocol was set up to simplify the interpretation of the data at the client side. The client is programmed in HTML5 or JavaScript using the Angular or Ionic framework. This framework ensures its adaptation at minor costs on different operating systems and allows access to the core functionalities of the devices.

#### Security Issues

The transition from laboratory to field test confronted us to questions regarding the access to the clinical data. As mentioned above, one of the priorities in developing our app was to provide efficiency and high usability. This implied that we needed to find a way to simplify the user authentication procedure without compromising overall security. Another constraint was the sharing of devices among the members of a nursing team (over 20 nursing staff per team), which therefore excluded the possibility of using biometric authentication methods. The most sensitive question was how to restrict access to authorized information in case of theft of the device.

**Figure 6:**
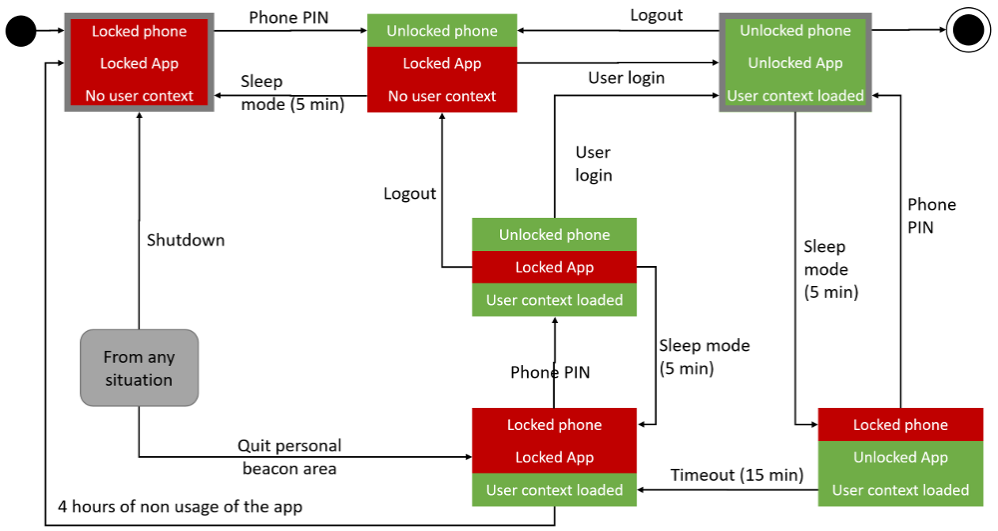
State diagram of the authentication process. Each state is composed of 3 substates: the phone (top line), and app (middle line) locking, as well as the user login (bottom line). PIN: Personal Identification Number.

Our current solution is the result of a Delphi process with the IT security experts of our institution. We developed a solution with a combination of Personal Identification Number (PIN) code, institutional password, and location beacons (Figure 6). 

**Table 3:** Participant demographics (N=10).

Characteristics		Statistics, n (%)
**Age (years)**		
	21-30	3 (30)
	31-40	5 (50)
	41-50	2 (20)
**Gender**		
	Female	6 (60)
	Male	4 (40)

At the initial state, the phone is locked, and no one is logged into the app. The user has to first unlock the phone using a PIN that is common to all devices for a ward. This PIN prevents access to the device in case of theft, as it is usually the case for every smartphone. As the PIN is shared between all ward users, it is not sufficient to ensure a personal access to the app. To ensure this personal access, each user has to log into the app using their institutional password, which associates a user identity to its authorization. Once logged in, the user can access clinical information. In the case of sleep mode, the phone gets locked, but the app does not lose its context. The user must then unlock it using the device PIN to get back to the previous context. If the phone remains unused for a longer period (>15 min), the app is locked; this means that the user has to enter both the PIN and their institutional password to retrieve their session. The user context remains loaded until the user is logged off by choice or if the app remains unused for more than 4 hours. In all cases, if the phone leaves the ward area covered by beacons, the app logs off and the phone gets locked. The user must then restart the whole authorization process and reset its context.

#### Functional Evaluation

Functional evaluation consisted of setting a list of likely use cases, which allow the tester to assess the proper behavior of the apps. Each functional test was composed of an objective, a precondition, a list of execution stages, input data, expected results, actual results, test report, and name of the testers. We ended up with 15 different tests going from user identification, patient identification, interactions with interventions, and so on.

### Evaluation

#### Usability Test

**Figure 7:**
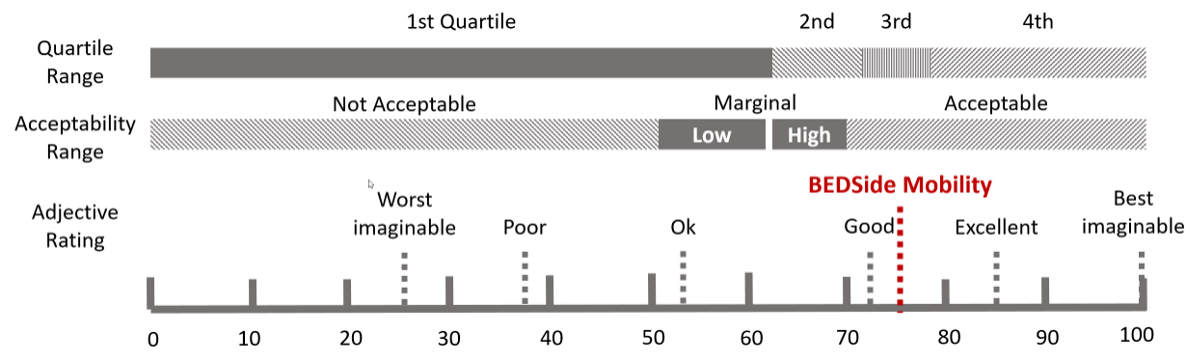
The system usability scale score of the Bedside Mobility app.

A total of 10 volunteer nurses from medicine and surgery wards took part in the usability test (Table 3). Overall, user satisfaction with the app was high, with a SUS of 75 (SD 16.5), as presented in Figure 7.

Overall, 9 out of 10 participants managed to accomplish the 10 required tasks, even though some tasks took more time for some users. Table 4 provides an overview of the difficulties encountered.

The most difficult tasks for the participants were the review of interventions, canceling an intervention, and the validation of PRN meds.

Overall, 1 user encountered several difficulties. The user completed 3 of the tasks without help, but had navigation issues and had trouble understanding the icons in the app. The interpretation of certain icons was also reported as a source of error or delay among other participants. Moreover, two of the other participants made errors during the test by clicking on the wrong buttons, but spontaneously corrected them. All potential sources of error or delay were revised after completing the testing, and the project team tested the revised version of the app.

**Table 4:** Identified shortcomings and their correction measures.

Identified shortcomings	Correction measures
Miscomprehension of the clinical scale icon	Identification with the users of a more appropriate icon to represent clinical scale
Unexpected navigation of the back button when an intervention is open	Modification of the navigation mechanism by closing the intervention when opened rather than returning to the previous page
Canceling the validation of an intervention	Improved explanations before app use can help avoid this confusion
Inconsistent implementation of the functional design validating the administration of a pro re nata drug	Integration of similar validation mechanism to administer PRN drug using consistent icons

#### Supervised Field Testing

Supervised field testing took place after the second cycle of development. The local ethical committee exempted this study of authorization due to its nature (quality improvement project) and the absence of nominative data collection. Overall, 1 investigator conducted daily observations over 2 weeks of about 5 different nurses per ward to see how they interacted with the tool. This test underlined the necessity to train the users adequately and to explain the concepts underlying the app (ie, it was not intended to replace the laptops on the WOWs) and helped to identify various bugs and inconsistencies, thus driving small improvements. After each field test, the bugs were fixed and the evolutions were integrated into preparation of the next field test.

#### Pilot Test in the Wards

**Figure 8:**
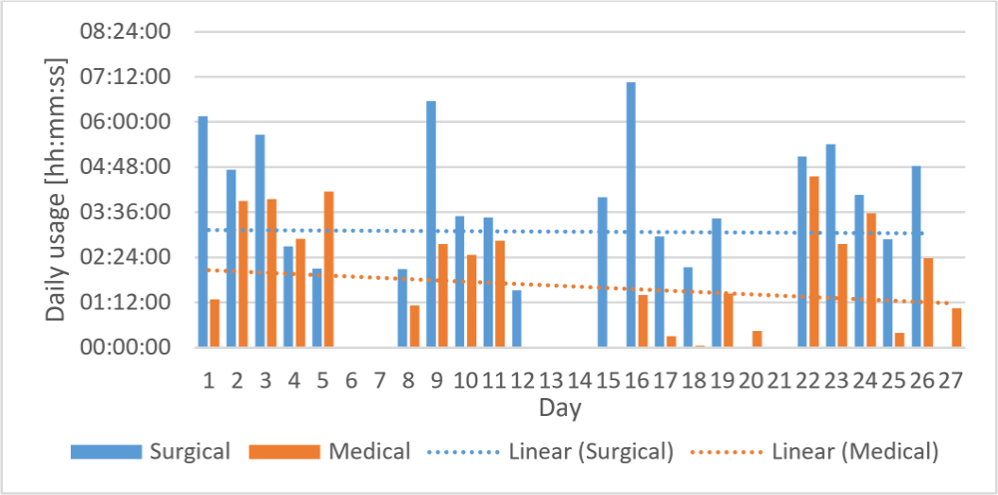
Daily usage per ward.

The study in the surgical unit took place for 5 weeks in July 2017 in the surgical ward and for 5 weeks in August 2017 in the medical ward. The first week was used to collect baseline data of workflow and EHR documentation. The fifth-week observations also included app use. During the study period, 27 nurses and nursing assistants used the app 427 times in the surgical ward and 23 participants used the app 239 times in the medical ward (Figure 8).

Participants were asked to verify their data entry before ending their shift to ensure the quality of clinical documentation. We explained that this was an initial pilot test with uncertain future, as the results would allow the hospital policy makers to decide whether to pursue the deployment of mobile devices.

#### Technology Acceptance

Our tailored UTAUT questionnaires were completed on a voluntary basis. In total, in the 2 wards, 16 care providers (8 in each ward) responded to the questionnaire. Responders were aged between 25 and 58 years (mean age 37 years). Table 5 provides a summary of the UTAUT results.

The questionnaire results revealed that the users considered the app easy to use, with a mean score of 5.6 for the effort dimension. The promotion of the app usage by the institution was evaluated as satisfactory, with a mean of 5.4. In terms of motivators of app use, the influence of the hierarchy for app use was clearly present (average of 5.9), whereas the influence of coworkers was much lower (average of 3.9).

**Table 5:** Mean and SD in the 5 dimensions of our tailored unified theory of acceptance and use of technology questionnaire (7-point Likert scale).

Measure	Statistics, mean (SD)
Performance	3.7 (1.8)
Effort	5.6 (1.4)
Influence	5.4 (1.3)
Condition	4.7 (1.7)
Intention	4.3 (2.3)

The conditions facilitating the usage were also considered adequate with an average score of 4.7. The weakest score was for the performance dimension, with a mean score of 3.7. The question “Do you think the BEDside app increased your productivity?” received the lowest score, with an average of 3. Intention to use the app in the future obtained an average score of 4.5.

## Discussion

### Use of Software Development Life Cycle Model

This study presents the development lifecycle of an app to support nursing bedside care, guided by an SDLC model that is adapted to the complexity of a health care setting. The strengths of this model are the strong involvement of end users throughout the process as well as the increasing ecology of subsequent evaluation stages. Gathering information from the end users helped to identify weaknesses in the workflow process and opportunities for supportive tools. The feasibility assessment not only involved technical issues within the EHR but also human resources, potential sources of funding, and acceptability from the users. Involving clinicians from the onset of the project helped engage them throughout the process, allowing them to make suggestions and understand feasibility and funding issues. Ultimately, we also needed to consider the potential benefits and deployment at an institutional level.

### Iterative Interactions With End Users

As already reported in the literature, involving users throughout the design and development process allowed them to better understand the possibilities and constraints for the app and ensure a good adoption [42-45].

The technical team also benefited from the proximity with end users, as it allowed them to suggest and develop functionalities that corresponded better to the workflow or to usual practice. This was in contrast to the interactions with the technical team for the EHR system. End users described frustrations with long-awaited “big” EHR improvements and subsequently did not always take the time to report the “small problems” to the technical support team. Communication between the end users and the developers was facilitated through frequent planned sessions as well as through the clinical team members who spent considerable time in the wards collecting information or testing functionalities with the users. Having this close and facilitated proximity between the users and the developers was therefore greatly beneficial to both parties.

### Heterogeneity of the Workflow

There is a strong variability in the nursing workflow between medical and surgical wards and even between the different surgical specialty teams (orthopedics, plastic surgery, etc). For example, differences lay in the patient monitoring and wound care, which involved more equipment in the surgical wards than in the medical wards. Integrating nursing representatives (directorate and head nurses) in the project team helped ensure that the specifications and functionalities designed were adapted to the nursing workflow, such as for clinical documentation, bedside tasks, and nursing handoffs.

### Use of an Institutional Device

In our setting, the institution provided devices to the nursing teams. The advantage of this solution is a more controlled and homogeneous environment, whereas disadvantages include higher costs due to the acquisition and maintenance of a large number of devices. Furthermore, having institutional devices provides an equitable access among health care providers, without discriminating against those who are not smartphone owners. Providing a device for each caregiver can be very costly; sharing the devices within the team may be more cost-effective. However, it requires a specific authentication policy that supports several users on a single device. This is complicated as smartphones are usually considered as personal devices and therefore have poor multi-identity management. Several solutions can be considered, but the final choice should carefully balance security and usability constraints. Indeed, authentication should not be too time-consuming as smartphone usage pattern is associated with a high number of authentications during daily usage [46]. A simple authentication is likely to improve user experience but may be insufficient for professional use in a health care environment with sensitive data [47-49].

### Appropriateness and Strengths of our Software Development Life Cycle Model

In health care environments, challenges to the success of HIT are largely due to nontechnical issues such as poor usability that impact communication and workflow. Therefore, involving end users all along the process is of vital importance. As most of the existing SDLC models are more focused on technical issues, we have defined our SDLC model to ensure end users’ participation in the early stage of the evaluation to maximize acceptance. The different stages of evaluation are designed to maximize the usefulness of the users’ involvement. Indeed, it is critical to make the best use of the care providers’ time in the implementation process because including participants is often difficult due to limited time. In addition, performing evaluations of growing ecological validity allowed us to minimize the risks associated with HIT in health care [50]. Indeed, interactions with medical data are never free of risks, and literature shows many examples of poor IT implementation that have worsened the care process [51-53]. By starting with a low ecological validity evaluation, our model helps resolve many issues at an early stage without the risk of compromising the integrity of data [54]. Indeed, the usability test helped us to detect some design problems that could have been considerable sources of errors in the field. Then, by performing limited tests under supervision, we ensured that the app was also adapted in real conditions, particularly for the existing workflow. Finally, the on-site evaluation is indispensable to ensure the acceptance of the app in real conditions and provided information about the care providers’ resistance as well as the organizational problems that can hinder the deployment of the app at a larger scale.

It is worth noting that even though end users were involved early in the process, the perception of the app productivity remained pretty low. This may be due to the instructions provided to the caregivers during the study. Indeed, as data manipulations undertaken through the app could have potential repercussions on the patients’ safety, we asked the participants to check the correct action or entry of the data in the EHR at the end of each shift. Therefore, care providers may have had the impression of having to repeat their work and thus had a poor perception of productivity. However, as the SDLC model optimizes the use of resources, minimizes risks, and maximizes acceptance, we can confidently recommend the use of our SDLC model in other medical settings.

### Limitations

We acknowledge that health care settings, needs, and workflow can differ considerably and that our study was conducted in a single institution. Therefore, generalizability to other health care setting may be limited. The EHR system is also home-grown and also limits generalizability in terms of feasibility, cost, and complexity assessments.

We were also particularly fortunate to have support from both top- and bottom-level stakeholders, who were involved at all stages. We emphasize the importance of user involvement and feedback in iterative cycles throughout the process to help ensure that the design and development are as tailored to the needs and workflow as possible.

### Conclusions

Knowledge about design and development of mHealth interventions is often scattered in the literature. In this study, we aimed to present the design and development of an mHealth intervention for caregivers according to a longitudinal methodology that ranges from the initial requirement identification process to the final product, with iterative development and testing processes. To the best of our knowledge, this paper is one of the first to describe the full process of design and development of an app in hospital settings using an SDLC model and to report its benefits and limitations. Each step of this process is necessary to ensure the creation of a useful and effective app that can truly support user needs, within a given workflow process. Although a close collaboration with clinicians throughout the entire project facilitated the development of a tailored solution, it was also important to involve all stakeholders, in particular, the IT security officers. In the health care setting, ensuring the adoption of an IT tool requires a solution that addresses the strong security constraints, while maintaining ease of use and good usability. Furthermore, we tried to anticipate how to potentially scale up this project to an institutional level, contingent on the results of the final testing phase.

## Abbreviations

EHR: electronic health record
HIS: health information system
HIT: health information technology
HUG: University Hospitals of Geneva
IT: information technology
IV: intravenous
mHealth: mobile health
PIN: Personal Identification Number
QR: Quick Response
REST: Representational State Transfer
SDLC: software development life cycle
SUS: System Usability Scale
UTAUT: unified theory of acceptance and use of technology
WOW: workstation on wheels
